# Association of multimorbidity patterns with potential out-of-hospital clinical service needs: results from a nationally representative sample of older Chinese

**DOI:** 10.3389/fpubh.2025.1586215

**Published:** 2025-08-26

**Authors:** Jing Yang, Jian Xiao, Zeyun Zhang, Jianlin Lin, Li Cao

**Affiliations:** ^1^School of Public Health, Hainan Medical University, Haikou, China; ^2^Nanjing Municipal Center for Disease Control and Prevention, Nanjing, China; ^3^Department of Science and Education, Xiang’an Hospital of Xiamen University, School of Medicine, Xiamen University, Xiamen, China; ^4^Faculty of Applied Sciences, Macao Polytechnic University, Macao, Macao SAR, China

**Keywords:** multimorbidity, health services, older adults, latent class analysis, network analysis

## Abstract

**Background:**

The rising prevalence of multimorbidity strains hospital-centric healthcare. Urgent attention is needed to understand potential out-of-hospital health service needs and inform policy for effective public health practices.

**Methods:**

Utilizing data from the China Health and Retirement Longitudinal Study (CHARLS), we first employed latent class analysis (LCA) to identify distinct patterns of multimorbidity. Subsequently, basic characteristics associated with each identified multimorbidity pattern were investigated using logistic regression models. Third, employing logistic mixed-effects models, we examined the associations between multimorbidity status, specific multimorbidity patterns, and Potential out-of-hospital clinical services need (POHCN). Fourth, network analysis was performed to explore the complex comorbidity network and identify central nodes within the patterns of multimorbidity. Finally, a stratified analysis by sex and age groups was conducted to examine the patterns and relationships between multimorbidity and POHCN across different sex and age categories.

**Results:**

Incorporating 11,215 participants aged 45 and above, with 51.4% being women, our study employed latent class analysis to delineate four latent patterns for 13 chronic diseases: “Kidney arthritic” (20%), “Lung-stomach disorder” (58%), “Asthma pattern” (5%), and “Multisystem pattern” (17%). Participants with multimorbidity exhibited a heightened potential demand for out-of-hospital care (OR = 2.53, 95% CI: 2.17–2.96). Notably, the “Multisystem pattern” displayed the highest demand (OR = 3.93, 95% CI: 3.23–4.79), followed by “Kidney arthritic” (OR = 3.50, 95% CI: 2.56–4.78), “Lung-stomach disorder” (OR = 3.09, 95% CI: 2.48–3.86), and “Asthma pattern” (OR = 2.07, 95% CI: 1.77–2.43). These associations persisted across diverse age groups (45–59, 60 + years). The results of the sex measurement uncertainty analysis indicated that the sex index adheres to the principles of measurement uncertainty. Network analysis identified heart disease, memory-related disease, and heart as pivotal nodes in the comorbid network. Furthermore, stratified analysis revealed statistically significant heterogeneity in the association between multimorbidity and POHCN across different sex and age groups.

**Conclusion:**

This study links multimorbidity to potential out-of-hospital medical service needs, identifying crucial diseases in the network. Crafting effective medical policies necessitates aligning clinical and public health practices with the characteristics of multimorbidity and its pivotal diseases.

## 1 Introduction

China’s aging population presents substantial challenges to its healthcare system. The transition of chronic diseases among older from single diseases to multimorbidity has led to a rapid surge in demand and expenditure for medical and health services (1, 2). Simultaneously, the escalating shortage of hospital beds and medical staff has become increasingly pronounced, posing significant challenges to China’s medical and health system (3, 4). The traditional hospital-centered medical service system proves inadequate in addressing the needs of the aging population in managing chronic diseases (5, 6). Moreover, the imbalance between the supply and demand for medical services may result in delays in emergency medical treatment, heightened patient mortality risks, reduced satisfaction levels for both doctors and patients, and an increase in the cost of medical services (7). Additionally, the frequent occurrence of public health emergencies, such as the COVID-19 pandemic, urgently compels China to explore new medical service models, giving rise to out-of-hospital clinical services (8). Hence, there exists a pressing imperative for China to comprehensively assess the prevailing demand for out-of-hospital clinical services and promulgate novel public health policies to steer medical and healthcare practices.

Out-of-hospital encompasses the provision of hospital-grade medical care delivered in non-hospital settings as an alternative to traditional inpatient clinical services. This includes various modalities, such as community-based care, home visits or telemedicine, home care programs, early discharge programs, clinical unit models, and other configurations, delivered in settings other than conventional hospital outpatient clinics, emergency departments, or primary care facilities (9, 10). The continual evolution of technology, artificial intelligence, and telemedicine has progressively broadened the scope of out-of-hospital clinical services. The development of out-of-hospital clinical models carries significant clinical and public health implications. From a clinical perspective, out-of-hospital clinical demonstrates noteworthy advantages in enhancing patients’ health quality and mitigating medical costs. A conducted study revealed that patients subjected to home remote monitoring exhibited lower hospital admission rates (38% versus 54.4%, *p* < 0.001) and mortality rates (17.2% versus 25.3%, *p* < 0.031) in comparison to those with chronic heart failure receiving traditional clinical services (11). Furthermore, the implementation of the home-based hospital model, as pioneered by Johns Hopkins University, indicated that patients receiving home-based medical services incurred an average of 19% lower care costs and reported higher satisfaction levels compared to counterparts with similar disease severity who underwent traditional hospitalization (12). This indicates that outpatient clinical services reduce costs compared to traditional inpatient care models. Additionally, out-of-hospital clinical services play a pivotal role in addressing the issue of disparate medical resource distribution and enhancing medical service accessibility, particularly for patients facing transportation and scheduling challenges (13).

In China, a substantial prevalence of multimorbidity has been documented among individuals aged 45 and above, reaching 56.73% (9). Given the chronic nature of these diseases, characterized by non-severity, prolonged duration, and challenging curability, the concentration of out-of-hospital medical services is directed toward the cohort of middle-aged and older individuals experiencing multimorbidity. Technological advancements, encompassing the Internet, digital medical technology, artificial intelligence, and the Internet of Things, have propelled the rapid evolution of out-of-hospital medical services. This evolution holds the promise of delivering more convenient medical interventions for the management of chronic diseases prevalent among the middle-aged and older demographic. Nevertheless, the trend of demand for out-of-hospital medical services among the middle-aged and older demographic in China remains uncertain. Clarifying this demand assumes paramount significance as it informs the establishment of a robust support system and the formulation of corresponding policy measures. Furthermore, a notable dearth of studies exists to scrutinize the determinants of out-of-hospital medical services, particularly the ambiguous association with multimorbidity. This lack of understanding hampers efforts to delineate the focal points for constructing an effective out-of-hospital medical service system.

To bridge this gap in understanding the landscape of potential out-of-hospital clinical service needs in China, this study employed nationally representative survey data targeting middle-aged and older individuals. The primary objectives included (1) evaluating the current status of potential out-of-hospital clinical service needs among the Chinese population aged 45 and above, (2) assessing the correlation between multimorbidity and the demand for potential out-of-hospital clinical services, and (3) identifying pivotal diseases in the context of comorbidities and delineating key areas for disease management within the framework of potential out-of-hospital clinical services.

## 2 Methods

### 2.1 Data source

The data originated from the China Health and Retirement Longitudinal Study (CHARLS), which is a nationally representative longitudinal survey among Chinese people aged 45 years or above. Probability Proportional to Size sampling procedure was conducted to investigate the association among health, economic status, and quality of life as aging. This study collected data through in-person interviews using a standardized questionnaire, covering 28 provinces in China. More detailed information about the CHARLS was present in previous studies (10–12). A total of 11,215 participants aged 45 years or above in the 2011, 2013, and 2015 waves were included in this work.

### 2.2 Measures

#### 2.2.1 Multimorbidity

Multimorbidity was measured by 13 self-rated chronic diseases: hypertension, dyslipidemia, diabetes, chronic lung diseases, liver disease, heart disease, stroke, kidney disease, stomach disease, emotional problems, memory-related diseases, arthritis, and asthma. (Supplementary Table 1) (13) If individuals reported more than two chronic diseases, they were considered to have multimorbidity.

#### 2.2.2 Potential out-of-hospital clinical services need

Potential out-of-hospital clinical service needs (POHCN) contained healthcare demands and unmet needs. Of which, demands were assessed by “Did you visit a hospital or be visited by a health worker or doctor for outpatient care in the last month”, and unmet needs were assessed by “What is the main reason for not seeking medical treatment”. Individuals were considered to have potential out-of-hospital clinical services needs if they reported “yes” in question 1 or “no time”, “inconvenient traffic”, or “poor hospital service” in question 2.

#### 2.2.3 Covariates

Building on Andersen’s behavioral model (14), covariates assessed in baseline were divided into three broad categories: predisposing [age (45–59, 60 years+), sex (male, female), education (no formal education, primary school, middle school or above), marital status (cohabitation, single), smoke (no, yes), drink (no, yes), exercise (no, yes), BMI (<18.5 kg/m^2^, 18.5–22.9 kg/m^2^, ≥ 23 kg/m^2^)], enabling [annual household expenditures (quartiles), public health insurance (no, yes), private health insurance (no, yes), residence (urban, rural)], and need factors [disability (no, yes)].

### 2.3 Statistical analyses

The chi-square trend test scrutinizes the annual increment in POHCN across distinct demographic populations. First, latent class analysis (LCA) was conducted to identify the latent patterns associated with 13 chronic diseases. LCA is a statistical approach specifically designed to handle categorical variables, making it particularly well-suited for analyzing comorbidity data, which often involves multiple outcomes. In contrast, conventional clustering methods like K-means are primarily aimed at continuous data and often face challenges in dealing with categorical variables, typically requiring additional preprocessing steps and demonstrating suboptimal performance. Therefore, it is frequently employed in an approach used to identify different subgroups within populations that often share similar response patterns (15). The best-fitting model was tested by conducting LCA for 1–7 classes. There were five statistical criteria to evaluate model fit: Akaike’s Information Criterion (AIC), adjusted Bayesian Information Criterion (aBIC), Bayesian Information Criterion (BIC), consistent Akaike’s Information Criterion (cAIC), and Entropy (16). The lower the value of statistical criteria, the better the model fit. Next, to verify the robustness of the latent classes, we conducted tests for measurement invariance by sex, and the primary dataset was partitioned into a training set (80%) and a testing set (20%). Measurement invariance refers to the consistency of measurements obtained for the same construct across different conditions (e.g., different groups, etc.). Third, we looked at the basic characteristics of various multimorbidity groups using logistic regression models. Fourth, network analysis was done to identify the structure representing relationships between 13 chronic diseases. Analyzing chronic networks helps identify the most influential chronic diseases among Chinese residents aged 45 and older. The network analysis approach is applied to data with dichotomous variables. Within this network, nodes represent 13 chronic diseases, and edges denote the strength of comorbidity association between these diseases (weighted). The weight values for these edges were estimated using regularized logistic regression. Identifying significant nodes within the chronic disease network is based on four centrality measures: Strength, Closeness, Betweenness, and Expected Influence (EI). Specifically, Strength indicates the sum of the weights of the edges connected to a node, reflecting the overall level of association for that disease. Closeness assesses the average proximity of a node to all other nodes in the network. Betweenness quantifies the extent to which a node acts as a central intermediary or hub on the paths connecting other nodes. Expected Influence evaluates the total influence of a node, accounting for both positive and negative associations (17, 18). Fifth, the logistic mixed-effect model was used to measure the association between multimorbidity, baseline latent patterns of 13 chronic diseases, and potential out-of-hospital clinical service needs. Finally, to further assess differential effects within subgroups, we performed a stratified analysis based on age, sex, and *p*-values were adjusted using the Bonferroni method. All statistical calculations were done in R. LCA and network analysis were conducted by the “poLCA” package and the “bootnet” package, respectively. The threshold for statistical significance was *p* < 0.05.

## 3 Results

### 3.1 Latent class analysis

**Figure 1:**
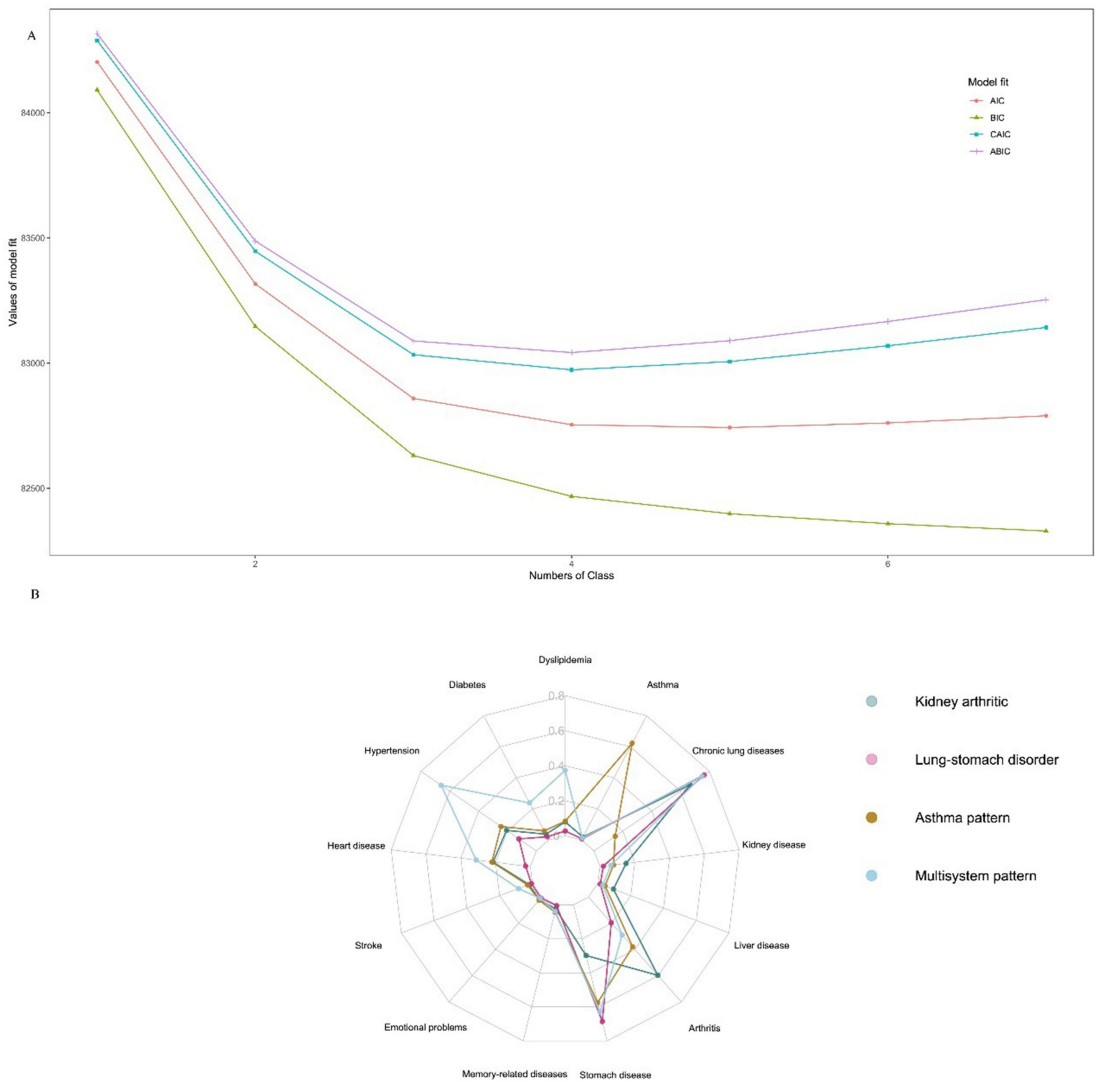
Latent class analysis for multimorbidity. **(A)** Model selection for latent class analysis. **(B)** Participants’ conditional probability of each chronic disease.

Supplementary Table 2 presents the fit statistics (BIC, aBIC, cAIC, AIC, and entropy) for the LCA models with one through seven latent classes. Given that the BIC is generally preferred for model selection with large sample sizes, the four-class model was selected as the final solution, as it yielded the lowest BIC value (82,973.41), and average posterior probability greater than 0.7, indicating a strong consistency of the members within the same cluster with their assigned categories (Figure 1A and Supplementary Table 2). Figure 1B illustrates the conditional probabilities of the latent class of multimorbidity for each chronic disease. Based on this, we summarize the characteristics of each latent class. Individuals in group 1 were more likely to experience kidney disease and arthritic disease. Thus, they were designated as “Kidney arthritic” (20%). Individuals in group 2 were more probably to have lung disease and stomach disease. Thus, they were described as “Lung-stomach disorder” (58%). Individuals in group 3 had the highest number of asthmatics. Thus, they were described as “Asthma pattern” (5%). Participants in Group 4 were at the highest risk for multiple diseases, including diabetes, hypertension, heart disease, dyslipidemia, and arthritis. Thus, they were tagged as “Multisystem pattern” (17%).

### 3.2 Robust evaluation of the LCA

First, Table 1 presents the results of measurement invariance (MI) testing across sex (male and female). We configured two models: Model 1 (the baseline model), allowing the item intercepts and loadings to vary freely between sexes, and Model 2 (the constrained model), which constrained these parameters to be equal across males and females. A comparison of model fit indices revealed that Model 2 (constrained) did not exhibit a significantly worse fit than Model 1 (freely estimated) (likelihood ratio difference test: ΔG^2^ = 895.44, df = 8,189, *p* > 0.05). These results support configural and metric/scalar measurement invariance across sex at the indicator level.

**Table 1:** Test of measurement invariance for latent classes between males and females.

Model	Log likelihood	DF	G^2^	AIC	BIC
Model 1: item-response probabilities are free to vary across the set	−41260.48	8,136	2084.82	2194.82	2597.7
Model 2: item-response probabilities constrained to be equal across sex	−41189.51	16,325	2980.26	3096.26	3521.11

G_2_^2^ – G_1_^2^ = 895.44, df = 8189, *P* > 0.05.

Second, consistent with our primary dataset, both the training and test sets retained an optimal four-class solution. The four-class model exhibited the lowest BIC values in both subsets (Training set: 66,090.85; Test set: 17,249.75) (Supplementary Tables 3, 4). Supplementary Figures 1, 2 present the conditional probabilities of the latent class of multimorbidity for each chronic disease within the main dataset, the training set, and the testing set, respectively.

### 3.3 Descriptive analysis

**Figure 2:**
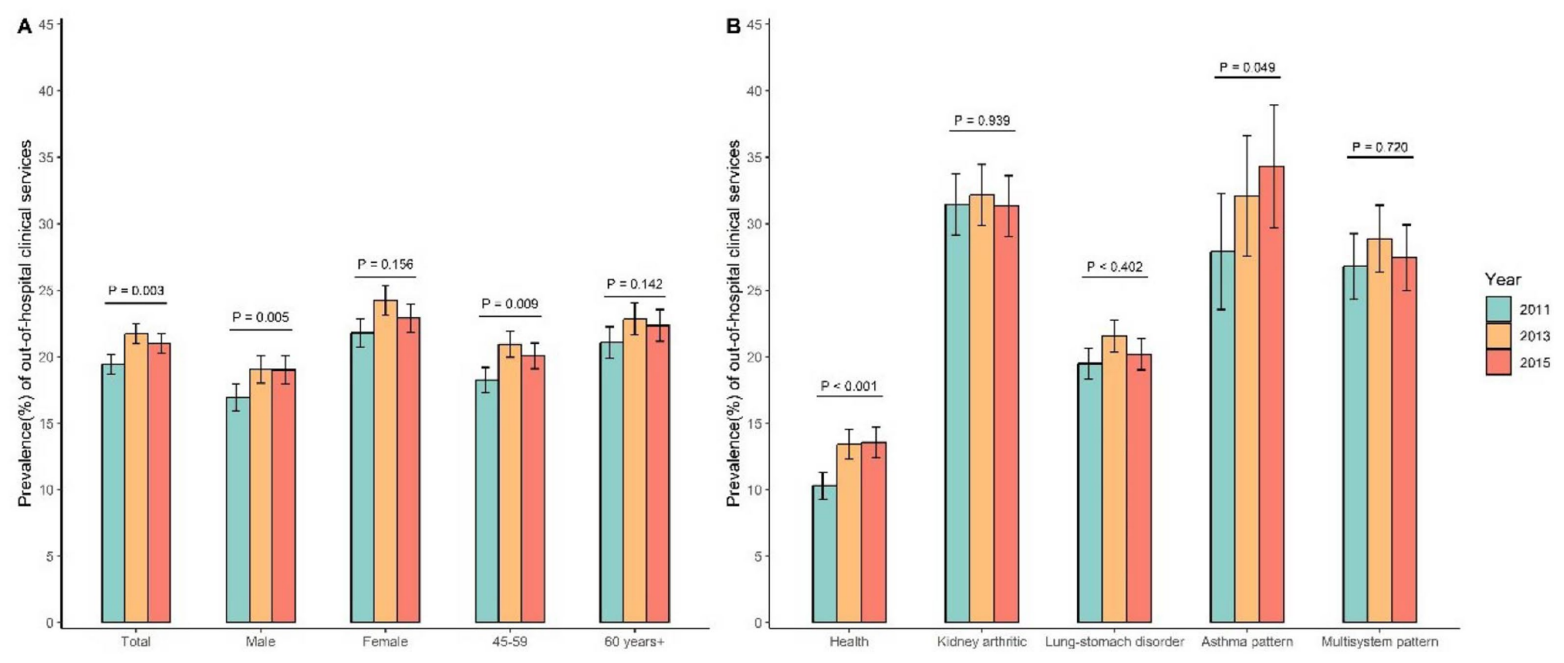
Trend of out-of-hospital clinical service needs among middle-aged and older people aged 45 and above in China, 2011–2015. **(A)** Trends in out-of-hospital clinical service needs grouped by sex and age. **(B)** Trends in out-of-hospital clinical service needs grouped by latent classes of multimorbidity.

In general, there exists an ascending trajectory in the demand for out-of-hospital medical services, particularly notable among men and individuals aged 45–59 years (Figure 2). Across different latent classes, the demand for out-of-hospital medical services exhibited an increasing trend in the healthy group, whereas the latent classes with multimorbidity groups did not register a direct increase. The “Kidney arthritic” class and “Asthma pattern” demonstrated relatively heightened out-of-hospital medical service needs, estimated at around 30% per year (Table 2). In a broader context, the four latent classes, when compared to the health group, were more likely to report disability, be 60 years old or older, female, possess lower educational attainment, and be single (Table 2).

**Table 2:** Sociodemographic characteristics of sample grouped by latent classes of multimorbidity.

Variables	Health (*N* = 3,482)	Kidney arthritic (*N* = 1,579)	Lung-stomach disorder (*N* = 4,496)	Asthma pattern (*N* = 406)	Multisystem pattern (*N* = 1,252)	Total (*N* = 11,215)	*P*-value
POHCN in 2011							
No	3,120 (89.6%)	1,081 (68.5%)	3,617 (80.4%)	292 (71.9%)	915 (73.1%)	9,025 (80.5%)	<0.001
Yes	358 (10.3%)	496 (31.4%)	874 (19.4%)	113 (27.8%)	335 (26.8%)	2,176 (19.4%)	
POHCN in 2013							
No	3,012 (86.5%)	1,070 (67.8%)	3,522 (78.3%)	275 (67.7%)	889 (71.0%)	8,768 (78.2%)	<0.001
Yes	466 (13.4%)	507 (32.1%)	969 (21.6%)	130 (32.0%)	361 (28.8%)	2,433 (21.7%)	
POHCN in 2015							
No	3,007 (86.4%)	1,083 (68.6%)	3,585 (79.7%)	266 (65.5%)	907 (72.4%)	8,848 (78.9%)	<0.001
Yes	471 (13.5%)	494 (31.3%)	906 (20.2%)	139 (34.2%)	343 (27.4%)	2,353 (21.0%)	
ADL							
Yes	3,352 (96.3%)	1,360 (86.1%)	4,124 (91.7%)	361 (88.9%)	1,093 (87.3%)	10,290 (91.8%)	<0.001
No	83 (2.4%)	205 (13.0%)	317 (7.1%)	42 (10.3%)	148 (11.8%)	795 (7.1%)	
Age							
45–59	2,341 (67.2%)	884 (56.0%)	2,577 (57.3%)	146 (36.0%)	578 (46.2%)	6,526 (58.2%)	<0.001
60 years+	1,141 (32.8%)	695 (44.0%)	1919 (42.7%)	260 (64.0%)	674 (53.8%)	4,689 (41.8%)	
Sex							
Male	1821 (52.3%)	605 (38.3%)	2,185 (48.6%)	250 (61.6%)	585 (46.7%)	5,446 (48.6%)	<0.001
Female	1,661 (47.7%)	974 (61.7%)	2,311 (51.4%)	156 (38.4%)	667 (53.3%)	5,769 (51.4%)	
Education							
No formal education	878 (25.2%)	489 (31.0%)	1,290 (28.7%)	104 (25.6%)	283 (22.6%)	3,044 (27.1%)	<0.001
Primary school	1,287 (37.0%)	676 (42.8%)	1827 (40.6%)	199 (49.0%)	504 (40.3%)	4,493 (40.1%)	
Middle school or above	1,317 (37.8%)	414 (26.2%)	1,379 (30.7%)	103 (25.4%)	465 (37.1%)	3,678 (32.8%)	
Marital status							
Cohabitation	3,143 (90.3%)	1,401 (88.7%)	4,008 (89.1%)	351 (86.5%)	1,112 (88.8%)	10,015 (89.3%)	0.168
Single	339 (9.7%)	178 (11.3%)	488 (10.9%)	55 (13.5%)	140 (11.2%)	1,200 (10.7%)	
Smoke							
No	2,222 (63.8%)	1,161 (73.5%)	3,024 (67.3%)	268 (66.0%)	940 (75.1%)	7,615 (67.9%)	<0.001
Yes	1,145 (32.9%)	386 (24.4%)	1,343 (29.9%)	126 (31.0%)	285 (22.8%)	3,285 (29.3%)	
Drink							
No	2,124 (61.0%)	1,150 (72.8%)	2,990 (66.5%)	267 (65.8%)	919 (73.4%)	7,450 (66.4%)	<0.001
Yes	1,354 (38.9%)	429 (27.2%)	1,503 (33.4%)	138 (34.0%)	331 (26.4%)	3,755 (33.5%)	
Exercise							
Yes	131 (3.8%)	48 (3.0%)	172 (3.8%)	21 (5.2%)	67 (5.4%)	439 (3.9%)	0.0488
No	1,238 (35.6%)	594 (37.6%)	1,653 (36.8%)	150 (36.9%)	453 (36.2%)	4,088 (36.5%)	
BMI							
<18.5	172 (4.9%)	109 (6.9%)	241 (5.4%)	48 (11.8%)	22 (1.8%)	592 (5.3%)	<0.001
[18.5–23)	1,371 (39.4%)	579 (36.7%)	1,638 (36.4%)	145 (35.7%)	196 (15.7%)	3,929 (35.0%)	
≥23	1,319 (37.9%)	616 (39.0%)	1821 (40.5%)	154 (37.9%)	766 (61.2%)	4,676 (41.7%)	
Annual household expenditures							
Q1	808 (23.2%)	327 (20.7%)	991 (22.0%)	100 (24.6%)	221 (17.7%)	2,447 (21.8%)	0.00287
Q2	745 (21.4%)	360 (22.8%)	962 (21.4%)	100 (24.6%)	253 (20.2%)	2,420 (21.6%)	
Q3	740 (21.3%)	380 (24.1%)	965 (21.5%)	79 (19.5%)	300 (24.0%)	2,464 (22.0%)	
Q4	668 (19.2%)	293 (18.6%)	921 (20.5%)	81 (20.0%)	284 (22.7%)	2,247 (20.0%)	
Public health insurance							
No	281 (8.1%)	87 (5.5%)	296 (6.6%)	21 (5.2%)	85 (6.8%)	770 (6.9%)	0.0107
Yes	3,186 (91.5%)	1,489 (94.3%)	4,181 (93.0%)	383 (94.3%)	1,160 (92.7%)	10,399 (92.7%)	
Private health insurance							
No	3,365 (96.6%)	1,533 (97.1%)	4,363 (97.0%)	398 (98.0%)	1,192 (95.2%)	10,851 (96.8%)	0.0261
Yes	93 (2.7%)	40 (2.5%)	99 (2.2%)	5 (1.2%)	47 (3.8%)	284 (2.5%)	
Residence							
Urban	1,210 (34.8%)	479 (30.3%)	1,608 (35.8%)	130 (32.0%)	626 (50.0%)	4,053 (36.1%)	<0.001
Rural	2,272 (65.2%)	1,100 (69.7%)	2,888 (64.2%)	276 (68.0%)	626 (50.0%)	7,162 (63.9%)	

### 3.4 Characteristics of the latent class of multimorbidity

Table 3 delineates the characteristics of distinct latent classes of multimorbidity. Individuals within the “Kidney arthritic” category exhibited a higher likelihood of disability, being over 60 years old, female, possessing primary school education, engaging in limited physical activity, and having a moderate-to-high income compared to the “Health” group. Those in the “Lung-stomach disorder” group were more prone to disability and being over 60 years old. Participants identified with the “Asthma pattern” were more likely to be over 60 years old, male, smokers, underweight, and with a lower income. In contrast, those in the “Multisystem pattern” group were more likely to experience disability, be over 60 years old, have less than a secondary school education, have higher body weight, and reside in urban areas.

**Table 3:** Sociodemographic characteristics of latent multimorbidity classes (Ref = “Health” class).

Variables		Kidney arthritic	Lung-stomach disorder	Asthma pattern	Multisystem pattern
Disability	Yes	Ref	Ref	Ref	Ref
	No	**5.29 (3.16–8.84, *p* < 0.001)**	**3.09 (1.93–4.94, *p* < 0.001)**	2.31 (0.96–5.59, *p* = 0.062)	**4.54 (2.47–8.33, *p* < 0.001)**
Age	45–59	Ref	Ref	Ref	Ref
	60 years+	**1.43 (1.10–1.85, *p* = 0.006)**	**1.52 (1.26–1.84, *p* < 0.001)**	**3.34 (2.19–5.11, *p* < 0.001)**	**3.46 (2.58–4.65, *p* < 0.001)**
Sex	Male	Ref	Ref	Ref	Ref
	Female	**1.62 (1.17–2.25, *p* = 0.004)**	1.20 (0.95–1.51, *p* = 0.132)	**0.48 (0.29–0.79, *p* = 0.004)**	1.22 (0.84–1.75, *p* = 0.292)
Education	No formal education	Ref	Ref	Ref	Ref
	Primary school	**1.38 (1.04–1.83, *p* = 0.025)**	1.29 (1.04–1.60, *p* = 0.020)	1.52 (0.93–2.47, *p* = 0.094)	**1.71 (1.19–2.46, *p* = 0.004)**
	Middle school or above	0.97 (0.69–1.36, *p* = 0.859)	0.96 (0.75–1.22, *p* = 0.746)	0.87 (0.48–1.58, *p* = 0.643)	**1.74 (1.16–2.60, *p* = 0.008)**
Marital status	Cohabitation	ref	ref	ref	ref
	Single	0.74 (0.49–1.11, *p* = 0.149)	0.89 (0.67–1.18, *p* = 0.407)	0.72 (0.36–1.42, *p* = 0.339)	0.92 (0.58–1.46, *p* = 0.718)
Smoke	No	Ref	Ref	Ref	Ref
	Yes	0.93 (0.67–1.28, *p* = 0.644)	0.94 (0.75–1.17, *p* = 0.569)	**0.53 (0.33–0.86, *p* = 0.009)**	0.81 (0.56–1.16, *p* = 0.247)
Drink	No	Ref	Ref	Ref	Ref
	Yes	**0.66 (0.49–0.88, *p* = 0.005)**	0.97 (0.79–1.18, *p* = 0.733)	0.67 (0.43–1.05, *p* = 0.084)	**0.66 (0.47–0.93, *p* = 0.017)**
Exercise	Yes	Ref	Ref	Ref	Ref
	No	**1.66 (1.06–2.60, *p* = 0.026)**	1.14 (0.85–1.53, *p* = 0.379)	0.99 (0.52–1.87, *p* = 0.972)	0.93 (0.60–1.43, *p* = 0.726)
BMI	<18.5	Ref	Ref	Ref	Ref
	[18.5–23)	0.68 (0.42–1.09, *p* = 0.113)	0.96 (0.66–1.38, *p* = 0.809)	**0.31 (0.17–0.58, *p* < 0.001)**	0.86 (0.42–1.78, *p* = 0.687)
	≥ 23	0.81 (0.50–1.32, *p* = 0.400)	1.28 (0.88–1.85, *p* = 0.199)	**0.50 (0.27–0.95, *p* = 0.033)**	**3.52 (1.73–7.14, *p* < 0.001)**
Annual household expenditures	Q1	Ref	Ref	Ref	Ref
	Q2	1.13 (0.82–1.55, *p* = 0.463)	0.93 (0.74–1.17, *p* = 0.521)	**2.03 (1.18–3.49, *p* = 0.010)**	1.13 (0.76–1.69, *p* = 0.549)
	Q3	**1.41 (1.02–1.96, *p* = 0.037)**	1.13 (0.89–1.43, *p* = 0.330)	1.85 (1.04–3.28, *p* = 0.035)	**1.70 (1.15–2.50, *p* = 0.007)**
	Q4	1.10 (0.77–1.56, *p* = 0.608)	1.06 (0.82–1.35, *p* = 0.669)	1.52 (0.82–2.82, *p* = 0.180)	1.49 (0.99–2.25, *p* = 0.057)
Public health insurance	No	Ref	Ref	Ref	Ref
	Yes	1.45 (0.87–2.41, *p* = 0.158)	1.13 (0.81–1.57, *p* = 0.481)	1.30 (0.57–2.95, *p* = 0.532)	1.62 (0.89–2.93, p = 0.113)
Private health insurance	No	Ref	Ref	Ref	Ref
	Yes	0.97 (0.43–2.16, *p* = 0.934)	1.05 (0.60–1.83, *p* = 0.863)	0.48 (0.06–3.76, *p* = 0.483)	1.26 (0.58–2.76, *p* = 0.562)
Residence	Urban	Ref	Ref	Ref	Ref
	Rural	1.24 (0.95–1.61, *p* = 0.108)	1.01 (0.84–1.22, *p* = 0.903)	1.10 (0.72–1.70, *p* = 0.653)	**0.69 (0.52–0.92, *p* = 0.013)**

*Bold indicates *P* < 0.05.

### 3.5 Association between multimorbidity and POHCN

**Figure 3:**
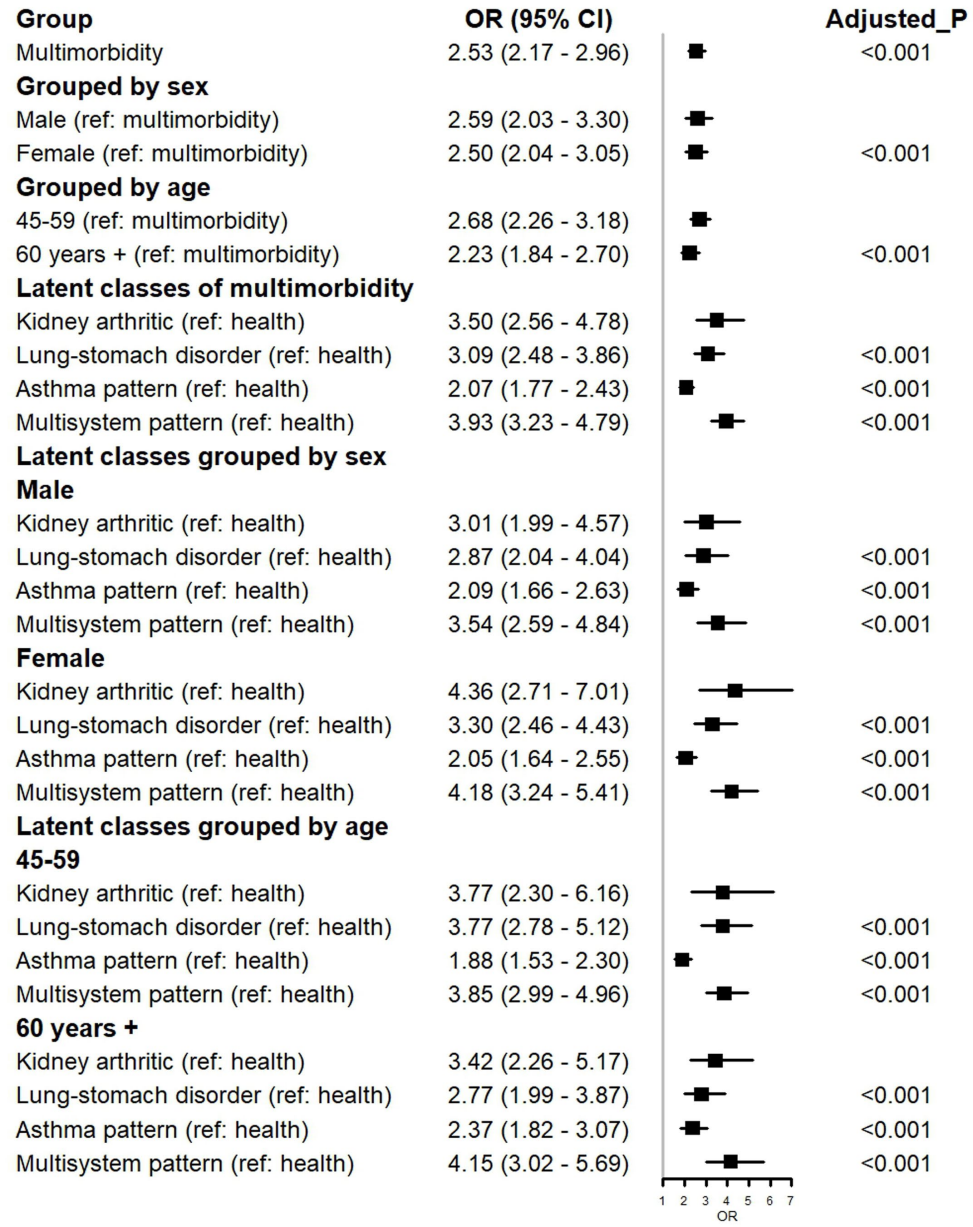
Forest plot for the association between out-of-hospital clinical service needs and multimorbidity.

Participants with multimorbidity exhibited notably elevated out-of-hospital medical service needs (OR = 2.53, 95% CI: 2.17–2.96) (Figure 3). This association was consistent across both male and female cohorts. While participants aged over 60 with multiple morbidities displayed a heightened demand for out-of-hospital health services, those in the 45–59 age group demonstrated an even greater need for such services.

In comparison to the healthy group, the “Multisystem pattern” cohort displayed the most substantial requirement for out-of-hospital medical services (OR = 3.93, 95% CI: 3.23–4.79), followed by “Kidney arthritic” (OR = 3.50, 95% CI: 2.56–4.78), “Lung-stomach disorder” (OR = 3.09, 95% CI: 2.48–3.86), and “Asthma pattern” (OR = 2.07, 95% CI: 1.77–2.43). The latent multimorbidity classes remained consistent, irrespective of grouping by male or 45–59 year, with the ranking of out-of-hospital care service needs being: “Multisystem pattern”, “Kidney arthritic”, “Lung-stomach disorder”, and “Asthma pattern”.

### 3.6 Stratified analysis of multimorbidity and POHCN

Figure 3 similarly presents the results of stratified analyses by sex and age. The results demonstrate that, compared with the healthy group, the “Kidney arthritic,” “Lung-stomach disorder,” “Asthma pattern,” and “Multisystem pattern” were consistently associated with an increased risk of POHCN across all sex and age subgroups. Specifically, within the male cohort compared to the healthy group, the Multisystem pattern exhibited the highest risk (OR = 3.54, 95% CI: 2.59–4.84, Adjusted *p* < 0.001), followed by the Kidney arthritic (OR = 3.01, 95% CI: 1.99–4.57, Adjusted *p* < 0.001). In the female cohort compared to the healthy group, the Kidney arthritic demonstrated the highest risk (OR = 4.36, 95% CI: 2.71–7.01, Adjusted *p* < 0.001), followed by the Multisystem pattern (OR = 4.18, 95% CI: 3.24–5.41, Adjusted *p* < 0.001). Among participants aged 45–59 years, compared to the healthy group, the Multisystem pattern conferred the highest risk (OR = 3.85, 95% CI: 2.99–4.96, Adjusted *p* < 0.001), followed by the Kidney arthritic and Lung-stomach disorder patterns. Among those aged ≥60 years, compared to the healthy group, the Multisystem pattern also presented the highest risk (OR = 4.15, 95% CI: 3.02–5.69, Adjusted *p* < 0.001), followed by the Kidney arthritic (OR = 3.42, 95% CI: 2.26–5.17, Adjusted *p* < 0.001). Notably, all adjusted *p*-values were less than 0.001.

## 4 Discussion

In the Chinese context, the conceptualization and application scenarios of out-of-hospital medical services have yet to be systematically investigated. This research pioneers an examination of the POHCN among the middle-aged and older population in China, utilizing comprehensive national longitudinal data. The analysis discerns an ascending trend in the POHCN. Furthermore, employing latent class analysis, the study explores multimorbidity patterns across 13 chronic diseases and scrutinizes their correlation with the POHCN. Additionally, network analysis is deployed to delineate the internal structural dynamics of these chronic diseases. The implications derived from this research bear significance in steering medical practices and formulating health policies tailored to the middle-aged and older demographic.

Numerous countries have initiated investigations into models of out-of-hospital medical services, commonly centered around family hospitals. Home-based medical services have emerged as a viable solution to alleviate the burden of medical care, concurrently enhancing the efficiency and quality of patient care (19). In the Chinese context, the predominant approach to addressing the healthcare needs of middle-aged and older individuals involves the integration of medical and older care alongside a hierarchical diagnosis and treatment system. Notably, a comprehensive out-of-hospital medical service system with a “community and family” core is yet to be established. China has begun to explore out-of-hospital medical care models, such as Internet medical services (20, 21). Our findings reveal a consistent annual increase in the demand for out-of-hospital medical services among middle-aged and older. Individuals with multimorbidity exhibit sustained high demand for medical and health services throughout the year. Consequently, we advocate for heightened attention from China’s health management authorities toward the establishment of a “community and family”-centric out-of-hospital medical service system. Emphasizing out-of-hospital medical services can catalyze enhancing primary healthcare capabilities, improving older health, addressing chronic diseases, and bolstering health emergency response capacities. Such strategic emphasis holds substantial significance in curbing medical and health service expenditures and elevating the quality of life for patients.

Our investigation identified four distinct multimorbid patterns, with the “Multisystem pattern” exhibiting the highest potential medical and health service needs, followed by the “Kidney arthritic” pattern, “Lung-stomach disorder”, and finally, “Asthma pattern”. Our findings align broadly with prior research and effectively delineate potential comorbidity patterns associated with category three or four chronic diseases (9). Patients characterized primarily by “Multisystem pattern” may exhibit a potential increased demand for outpatient healthcare services, which could stem partly from the need for weekly comprehensive whole-body examinations among some individuals. Conversely, others with multimorbidity patterns may not require frequent hospital-based care despite their multimorbidity diagnosis, necessitating only periodic monitoring of parameters including blood glucose, blood pressure, cardiac function, and hepatic and renal function. Research indicates that the demand for diagnosis, treatment, and management of chronic kidney disease patients in China remains unmet (22).

Similarly, the “Asthma pattern” exhibits a robust association with the POHCN. Conditions within this pattern, such as lung diseases and asthma, are marked by frequent exacerbations, likely contributing to heightened healthcare utilization (23). Despite 17% of participants having chronic diseases spanning multiple systems, the POHCN did not rank the highest. Notably, the “Lung-stomach disorder” pattern comprised a younger demographic (45–59 years) compared to other comorbid modalities, potentially encountering barriers to accessing comprehensive healthcare due to work-related constraints. Hence, tailoring out-of-hospital medical services according to the unique characteristics of each potential model holds paramount significance in enhancing primary healthcare for middle-aged and older individuals. This approach aims to improve overall quality of life, mitigate hospital admissions, and potentially prevent premature mortality. It holds paramount significance to elucidate the intricate relationship between multimorbidity and the potential demand for out-of-hospital medical services, paving the way for the establishment of a comprehensive healthcare system designed to cater to patients across their entire life cycle.

To enhance the understanding of the internal relationship between multimorbidity and identify intervention centers for multimorbidity, we performed a network analysis (Supplementary Figure 3A). The results of network analysis showed that heart disease, memory-related disease, and hypertension were the most important diseases in the disease network, as they were associated with other diseases the most (Supplementary Figure 3B). The Betweenness subgraph revealed a significantly higher occurrence of memory-related disorders compared to other conditions (evidenced by their location at the far right of the distribution). This positioning suggests that memory-related disorders may act as critical “hubs” within the comorbidity network, potentially bridging neurological disorders with various chronic diseases. Conversely, liver disorders, emotional problems, and gastric diseases exhibited the weakest correlations with other diseases, consistently ranking low across all measured metrics. This pattern indicates their relatively peripheral position in the network. Collectively, these findings underscore the importance of delineating distinct features within multimorbidity patterns using network analysis and of characterizing the specific roles individual chronic diseases play (e.g., as hubs or peripherals). Based on this network perspective, out-of-hospital healthcare services can be strategically deployed across five key dimensions: screening and diagnosis, intervention and treatment, monitoring and management, telemedicine models, and hospital-level patient services.

Various countries, including those in Europe and the United States, have initiated home hemodialysis treatments as early as the 1950s, achieving notable success in significantly mitigating travel inconveniences and infection risks for hemodialysis patients (24). Regarding the promotion of home dialysis, Cheetham MS et al. found that home hemodialysis (HD) significantly reduces long-term healthcare costs compared to in-center HD, yielding approximate annual savings of $15,000–25,000 USD per patient. Notably, substantial cost savings were particularly observed within insurance-payer systems. Furthermore, concerning AI-assisted monitoring, modeling based on UK NHS data demonstrates that an AI early warning system (integrated with wearable devices) reduces hospitalization rates for acute episodes and lowers associated costs (25–31). Similarly, China is poised to implement out-of-hospital medical services tailored to the characteristics of each multimorbidity pattern and focal chronic diseases. This includes offering home hemodialysis for patients exhibiting the “kidney arthritic” pattern, employing AI-assisted screening and intervention for those with the “Asthma pattern,” and facilitating out-of-hospital coagulation testing and blood glucose management for patients presenting the “multisystem pattern.”

In essence, the nascent developments in out-of-hospital medical services in China foreshadow a promising trajectory. Collaboration among the government, medical professionals, academia, and the medical industry is imperative to collectively construct a problem-oriented out-of-hospital medical service system that aligns with evolving healthcare needs.

The selection of the POHCN variable as a proxy indicator for unmet/practical need for out-of-hospital clinical services offers several advantages. First, self-reported unmet healthcare needs and healthcare utilization records in the CHARLS database inherently carry recall bias. Second, even when outpatient visit frequency is available in CHARLS, it is impossible to determine if subsequent traditional inpatient clinical services were forgone due to access barriers. As defined by responses to two specific questions (regarding POHCN), this variable directly captures unmet need.” While our study represents the initial exploration into the necessity for multimorbidity and out-of-hospital medical services, it is essential to acknowledge certain limitations inherent in this investigation. Firstly, the chronic disease data sourced from the CHARLS dataset relied on self-reported information, introducing the potential for recall bias or the presence of undiagnosed chronic diseases, thereby influencing the study outcomes. Secondly, the database utilized in this research did not encompass a comprehensive range of chronic diseases, potentially impacting the categorization of multi-incidence patterns. Thirdly, the assessment of the demand for out-of-hospital medical services relies on an indirect measurement approach, lacking direct measurement of specific needs for out-of-hospital medical care programs. Fourth, exploration of healthcare access or policy factors unmeasurable within the CHARLS database was limited. Nevertheless, steps were taken to partially mitigate the potential risk of bias stemming from this. Consequently, the measurements in this study may result in an underestimation of the genuine out-of-hospital medical service needs due to the absence of a direct evaluation of these needs.

## 5 Conclusion

This research offers novel insights into the POHCN among middle-aged and older exhibiting diverse multimorbidity patterns. It posits that distinct cohorts with varying multimorbid patterns present dissimilar susceptibilities and intensities concerning unplanned hospital care utilization. Specifically, middle-aged and older demonstrating patterns characterized by kidney disease, osteoarthritis, and asthma; as well as those with cardiac disease, memory-related disease, and hypertensive multimorbidity, emerge as pivotal focal points for the implementation of targeted public health intervention policies. The findings of this study may serve as valuable guidance for clinicians and policymakers, facilitating improved responsiveness to out-of-hospital medical care needs and enhancing the monitoring and care provision for older experiencing multimorbidity.

## Data Availability

Publicly available datasets were analyzed in this study. This data can be found here: https://charls.pku.edu.cn/.

## Supplementary material

Supplementary Figure 1 The conditional probability of participants in the training set being diagnosed with each chronic condition.

Supplementary Figure 2 The conditional probability of participants in the test set being diagnosed with each chronic condition.

Supplementary Figure 3 Network analysis of middle-aged and older people aged 45 and above in China. **(A)** Network structure diagram of 13 chronic diseases. The darker the color, the more relevant it is. **(B)** Evaluation index for network analysis.
