# Supplementary figures and images for: Association of multimorbidity patterns with potential out-of-hospital clinical service needs: results from a nationally representative sample of older Chinese
Source: Front Public Health. 2025 Aug 26;13:1586215. doi: 10.3389/fpubh.2025.1586215 (PMC12417407; doi:10.3389/fpubh.2025.1586215)

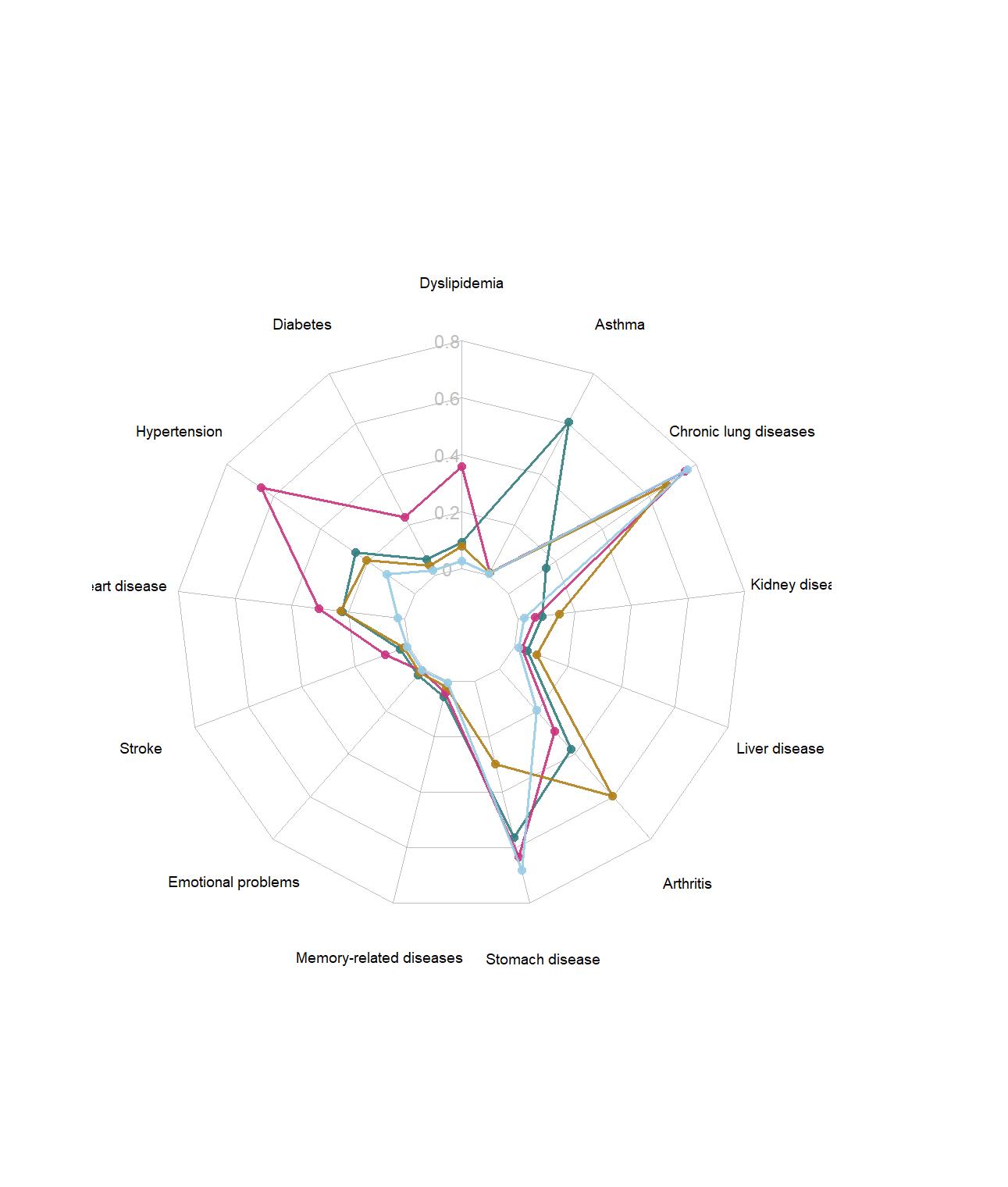

Supplement: Supplementary Figure 1 — The conditional probability of participants in the training set being diagnosed with each chronic condition. [file Image_1.jpg]

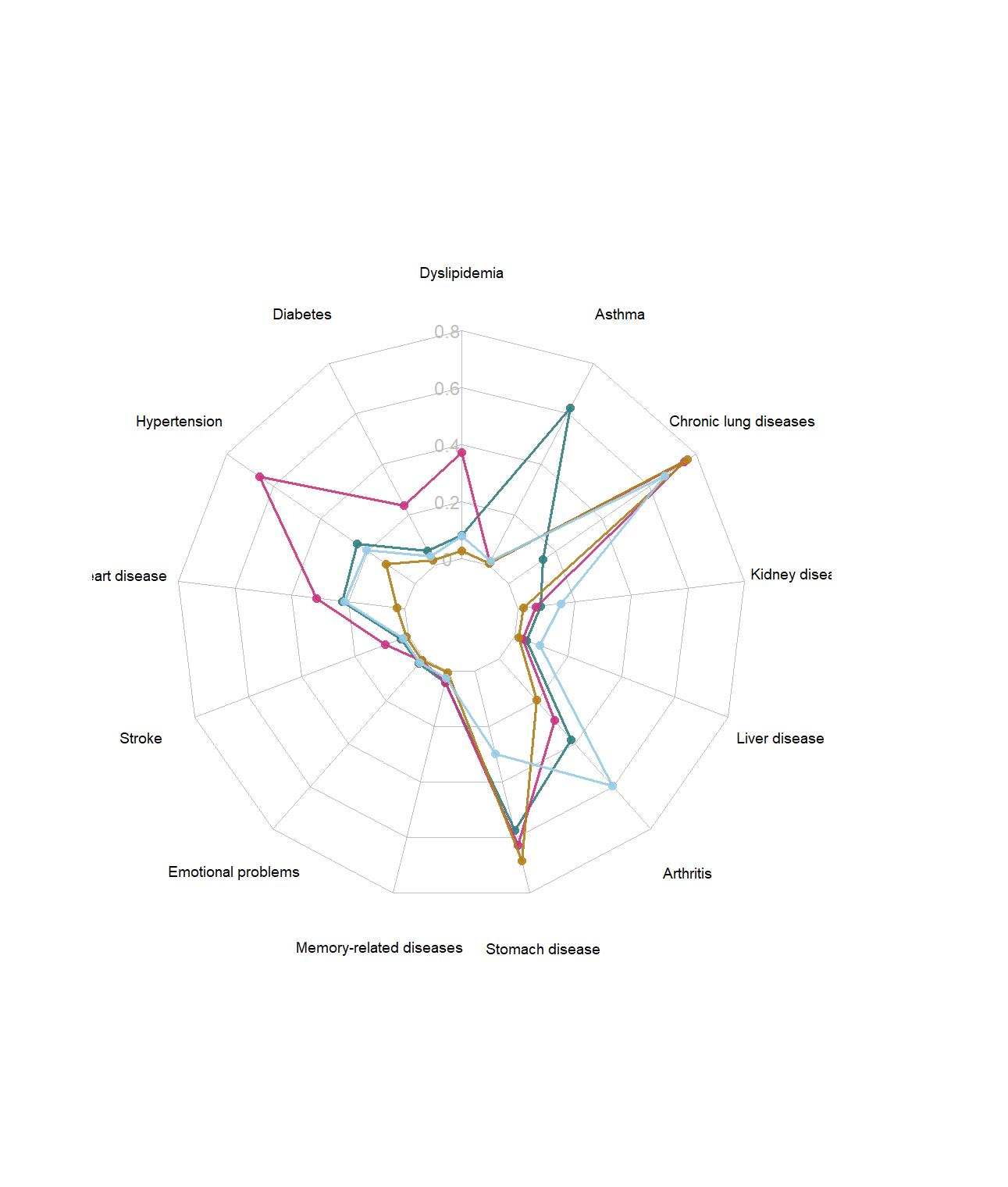

Supplement: Supplementary Figure 2 — The conditional probability of participants in the test set being diagnosed with each chronic condition. [file Image_2.jpg]

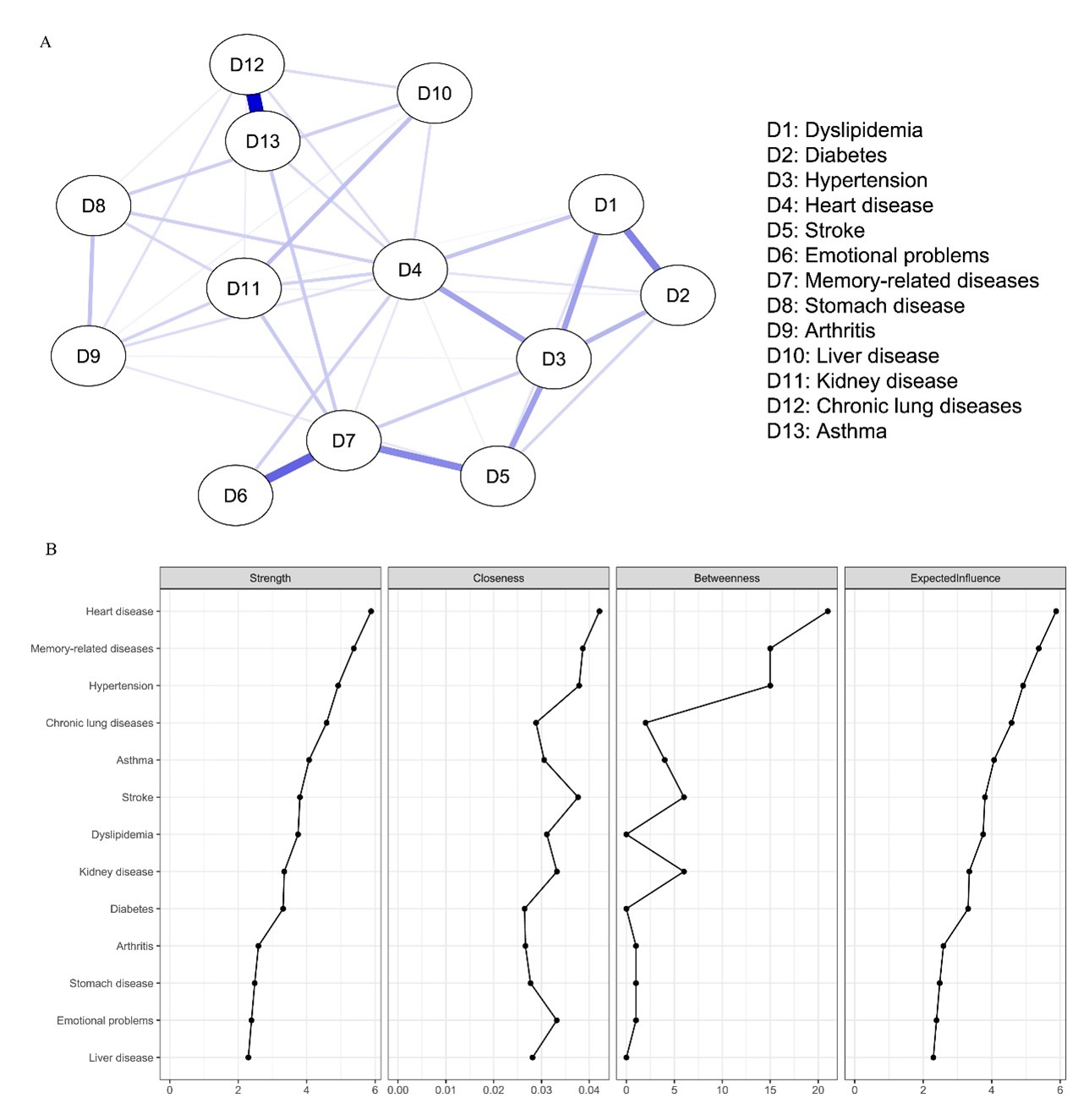

Supplement: Supplementary Figure 3 — Network analysis of middle-aged and older people aged 45 and above in China. (A) Network structure diagram of 13 chronic diseases. The darker the color, the more relevant it is. (B) Evaluation index for network analysis. [file Image_3.jpg]
