# Supplementary material for: Association of multimorbidity patterns with potential out-of-hospital clinical service needs: results from a nationally representative sample of older Chinese
Source: Front Public Health. 2025 Aug 26;13:1586215. doi: 10.3389/fpubh.2025.1586215 (PMC12417407; doi:10.3389/fpubh.2025.1586215)
Supplement: Supplementary file 4 [file Table_1.DOCX]

Supplementary Table 1 Thirteen chronic disease definitions in the CHARLS database

| Classification of diseases | Definition |
| --- | --- |
| Hypertension | Self-report of having been diagnosed with hypertension by a doctor. |
| Diabetes | Self-report having been diagnosed with diabetes or hyperglycemia by a doctor |
| Lung disease | Self-report having been diagnosed by a doctor with a chronic lung disease such as chronic bronchitis or emphysema (excluding tumors or cancer). |
| Heart disease | Self-report having been diagnosed with a heart attack, coronary heart disease, angina, congestive heart failure, or other heart problems by a doctor. |
| Stroke | Self-report of having been diagnosed with a stroke by a doctor. |
| Emotional problems | Self-report having been diagnosed by a doctor with any emotional, neurological, or psychiatric problems. |
| Arthritis | Self-report of having been diagnosed with arthritis or rheumatism by a doctor. |
| Dyslipidemia | Self-report having been diagnosed with dyslipidemia (elevated low-density lipoprotein, triglyceride (TG), and total cholesterol, or low HDL levels) by a doctor. |
| Liver disease | Self-report having been diagnosed by a doctor with any liver disease (except fatty liver, tumors, and cancer). |
| Kidney disease | Self-report having been diagnosed by a doctor with any kidney disease (other than tumor or cancer). |
| Stomach disease | Self-report having been diagnosed by a doctor with any stomach or other digestive disorder (other than a tumor or cancer). |
| Asthma | Self-report having been diagnosed with asthma by a doctor. |
| Memory-related diseases | Self-report of having been diagnosed with a memory-related disorder by a doctor. |
