# Supplementary material for: Association of multimorbidity patterns with potential out-of-hospital clinical service needs: results from a nationally representative sample of older Chinese
Source: Front Public Health. 2025 Aug 26;13:1586215. doi: 10.3389/fpubh.2025.1586215 (PMC12417407; doi:10.3389/fpubh.2025.1586215)
Supplement: Supplementary file 5 [file Table_2.DOCX]

Supplementary Table 2 Model Fit Statistics for Latent Class Analysis using the Complete Dataset

| Class | AIC | BIC | aBIC | cAIC | Entropy | Avepp |
| --- | --- | --- | --- | --- | --- | --- |
| 1 | 84091.41 | 84289.18 | 84203.38 | 84316.18 | 0.4581 | 1.0000 |
| 2 | 83146.72 | 83447.04 | 83316.75 | 83488.04 | 0.3375 | 0.8695 |
| 3 | 82630.96 | 83033.83 | 82859.05 | 83088.83 | 0.3112 | 0.8685 |
| 4 | 82467.99 | 82973.41 | 82754.14 | 83042.41 | 0.3551 | 0.8384 |
| 5 | 82398.58 | 83006.56 | 82742.79 | 83089.56 | 0.4324 | 0.7951 |
| 6 | 82351.85 | 83062.38 | 82754.13 | 83159.38 | 0.3832 | 0.7119 |
| 7 | 82326.66 | 83139.73 | 82786.99 | 83250.73 | 0.4175 | 0.6954 |

*AIC: Akaike's information criterion; BIC: Bayesian information criterion; aBIC: adjusted Bayesian information criterion; cAIC: consistent Akaike's information criterion; Avepp: Average posterior probability.
