# Supplementary material for: Association of multimorbidity patterns with potential out-of-hospital clinical service needs: results from a nationally representative sample of older Chinese
Source: Front Public Health. 2025 Aug 26;13:1586215. doi: 10.3389/fpubh.2025.1586215 (PMC12417407; doi:10.3389/fpubh.2025.1586215)
Supplement: Supplementary file 6 [file Table_3.DOCX]

Supplementary Table 3 Model Fit Statistics for Latent Class Analysis using the Test Set

| Class | AIC | BIC | aBIC | cAIC | Entropy | Avepp |
| --- | --- | --- | --- | --- | --- | --- |
| 1 | 66836.27 | 67028.02 | 66942.21 | 67055.02 | 0.4967 | 1 |
| 2 | 66144.18 | 66435.36 | 66305.07 | 66476.36 | 0.3682 | 0.8558 |
| 3 | 65749.44 | 66140.04 | 65965.26 | 66195.04 | 0.2986 | 0.8483 |
| 4 | 65600.82 | 66090.85 | 65871.58 | 66159.85 | 0.3110 | 0.8469 |
| 5 | 65552.56 | 66142.01 | 65878.25 | 66225.01 | 0.4199 | 0.8177 |
| 6 | 65517.67 | 66206.55 | 65898.30 | 66303.55 | 0.3724 | 0.7650 |
| 7 | 65493.89 | 66282.20 | 65929.46 | 66393.20 | 0.3735 | 0.7568 |

*AIC: Akaike's information criterion; BIC: Bayesian information criterion; aBIC: adjusted Bayesian information criterion; cAIC: consistent Akaike's information criterion; Avepp: Average posterior probability.
