# Supplementary material for: Association of multimorbidity patterns with potential out-of-hospital clinical service needs: results from a nationally representative sample of older Chinese
Source: Front Public Health. 2025 Aug 26;13:1586215. doi: 10.3389/fpubh.2025.1586215 (PMC12417407; doi:10.3389/fpubh.2025.1586215)
Supplement: Supplementary file 7 [file Table_4.DOCX]

Supplementary Table 4 Model Fit Statistics for Latent Class Analysis using the Training Set

| Class | AIC | BIC | aBIC | cAIC | Entropy | Avepp |
| --- | --- | --- | --- | --- | --- | --- |
| 1 | 17283.59 | 17537.91 | 17352.12 | 17464.91 | 0.4469 | 1 |
| 2 | 17040.06 | 17437.91 | 17144.14 | 17315.40 | 0.3159 | 0.8735 |
| 3 | 16935.39 | 17274.40 | 17075.01 | 17304.75 | 0.3144 | 0.8747 |
| 4 | 16925.15 | 17249.75 | 17100.30 | 17388.53 | 0.3451 | 0.8379 |
| 5 | 16921.13 | 17319.53 | 17131.82 | 17478.53 | 0.4222 | 0.8025 |
| 6 | 16915.77 | 17395.53 | 17161.99 | 17567.18 | 0.3929 | 0.7258 |
| 7 | 16922.51 | 17556.93 | 17204.27 | 17667.93 | 0.4708 | 0.6995 |

*AIC: Akaike's information criterion; BIC: Bayesian information criterion; aBIC: adjusted Bayesian information criterion; cAIC: consistent Akaike's information criterion; Avepp: Average posterior probability.
